# Development and psychometric testing of an instrument to compare career choice influences and perceptions of nursing among healthcare students

**DOI:** 10.1186/s12909-017-0910-7

**Published:** 2017-04-27

**Authors:** Sok Ying Liaw, Ling Ting Wu, Violeta Lopez, Yeow Leng Chow, Siriwan Lim, Eleanor Holroyd, Khoon Kiat Tan, Wenru Wang

**Affiliations:** 1 Alice Lee Centre for Nursing Studies, Yong Loo Lin School of Medicine, National University of Singapore, Level 2, Clinical Research Centre, Block MD11 10 Medical Drive, Singapore, 117597 Singapore; 2 Auckland University of Technology, 90 Akoranga Drive, Northcote, Auckland New Zealand; 3 School of Health Sciences, Nanyang Polytechnic, Singapore, Singapore

**Keywords:** Career choice, Healthcare course, Nursing recruitment, Psychometric testing, Scale development

## Abstract

**Background:**

With the availability of more healthcare courses and an increased intake of nursing students, education institutions are facing challenges to attract school leavers to enter nursing courses. The comparison of career choice influences and perception of nursing among healthcare students can provide information for recruitment strategies. An instrument to compare the influences of healthcare career choice is lacking. The purpose of this study is to develop and evaluate the psychometric properties of an instrument to compare the influences of healthcare career choice with perceptions of nursing as a career choice.

**Methods:**

The study was conducted in two phases. In phase one, two sets of scales with parallel items that measure the influences of healthcare career choice and perceptions of nursing as a career choice were developed through an earlier qualitative study, literature review, and expert validation. Phase two involved testing the construct validity, concurrent validity and reliability with a convenience sample of 283 first year healthcare students who were recruited at two education institutions in Singapore.

**Results:**

An exploratory factor analysis revealed 35-parallel items in a six-factor solution (personal interest, prior healthcare exposure, self-efficacy, perceived nature of work, job prospects, and social influences) that explained 59 and 64% of the variance for healthcare career choice and nursing as a career choice respectively. A high correlation (*r* = 0.76, *p* < 0.001) was obtained with an existing tool, confirming the concurrent validity. The internal consistency was sufficient with Cronbach’s alpha of 0.93 for healthcare career choice and 0.94 for nursing as a career choice. The test-retest reliability was acceptable with an Intraclass Correlation Coefficient of 0.63 for healthcare career choice and 0.60 for nursing as a career choice.

**Conclusions:**

The instrument provides opportunities for understanding the differences between influences of healthcare career choice and perceptions of nursing as a career choice. This comparative understanding of career choice influences can guide educator and policy-makers on nursing recruitment.

**Electronic supplementary material:**

The online version of this article (doi:10.1186/s12909-017-0910-7) contains supplementary material, which is available to authorized users.

## Background

The aging population leading to the expansion of healthcare infrastructure has contributed to an increase in global demands for healthcare professional workforce. A pool of health care professionals who are better prepared in caring for people with multiple chronic conditions are crucial in addressing current healthcare challenges [1]. A worldwide shortage of nurses has been reported [2, 3]. Compared with other healthcare courses, nursing courses have the largest recruitment target in order to meet the ever-growing demand for a nursing workforce. This recruitment target is met with threat from the increase intake of other healthcare courses.

School-age students are attracted to healthcare related courses due to career attributes such as altruism, job stability, financial remuneration, professional prestige, job autonomy and familial influences [4]. There are differences in factors influencing career choice among healthcare students. While the influence of altruism has a significant influence in the career choice for nursing [5], medicine [6] and pharmacy [7], it has less influence in dentistry [8]. The influence of financial remuneration was important in pharmacy [9] and dentistry [10], but less so in nursing [11] and medicine [6]. The influence of family appeared to be important on career choice in pharmacy [12] and medicine [6], while its influence on career choice in nursing and dentistry is inconclusive. As most of the existing studies examined the influences of career choice on a single healthcare discipline, future studies are needed to compare the influences of career choice between healthcare disciplines.

Among healthcare courses, nursing has been perceived as an unpopular choice and is seldom a first-preference career [13, 14]. With the availability of instruments to measure the perception of nursing, the views of school-age students were extensively explored [15–18]. Nursing was perceived by school-age students as less intellectual [19], having low job autonomy [20], involving too much hard work [21], holding ‘dirty’ work [22], and receiving low salaries [23]. Although the psychometric properties of the instruments used in these studies require a more in-depth evaluation [24], the outcomes lend further support to the belief that nursing as a career choice holds a low public image.

To further understand which career attributes of nursing were perceived as less ideal, an instrument known as the Indiana Instrument was designed to determine the differences in attitudes between ‘ideal career’ and ‘nursing as a career’. Using two sets of questionnaires with 17-parallel items, this tool was widely used to understand the difference between the ideal career and nursing career among school-age students [25–27]. A nursing career was found to match an ideal career in terms of ability to care for others, hard work, busyness and job security [25, 27]. A study by Cohen et al. [25] reported that nursing was less than ideal in terms of respect, appreciation, autonomy and financial remuneration. An earlier study by Mendez and Louis reported nursing career to fall short of an ideal career in terms of knowledge, power and job stability [26].

The utility of the existing instruments has primarily focused on school students’ perception of nursing as a career. It appears to be more important to target on students who are interested in healthcare career, as these students often chose a particular healthcare career after consideration of related health profession. However, existing instruments do not allow for comparison of influences of healthcare career choice with the perception of nursing. There is a need to examine why those who are inclined to choose healthcare courses eventually did not select a nursing career [28]. The comparison of career choice influences and perception of nursing as a career choice among healthcare students would highlight the comparatively stronger attractive factors, which could be used to identify specific recruitment strategies to attract student to choose nursing. Prior to conducting the study, there was a need to develop and evaluate the psychometric properties of scales to compare the influences of healthcare career choice with perceptions of nursing as a career choice.

## Methods

A two-phase prospective study was conducted. Phase 1 included the development and content validation of the instrument, (1) Healthcare Career Choice (HCC) scale and (2) Nursing Career Choice (NCC) scale, and phase 2 involved the psychometric evaluation of the instrument.

### Phase 1: Development and content validation of the scales

#### Subscale specification

A qualitative exploratory descriptive study was conducted earlier to explore factors influencing career choice and perception of nursing among healthcare students. The study was conducted with 59 first-year healthcare students from three higher education institutions undertaking health-science related courses including dentistry, dental hygiene, medicine, nursing, pharmacy, physiotherapy and occupational therapy through eight focus group discussions. Each focus group, consisted of six to eight participants and lasted about 60–75 min. Six themes emerged from the thematic analysis: (1) personal interest, (2) prior healthcare exposure, (3) academic performance, (4) perceived nature of work, (5) job prospects, and (6) social influences [29]. These themes were identified as subscales for the HCC-NCC instrument.

#### Item development

The Indiana Instrument was the most widely used instrument for determining the differences of career attributes or characteristics between nursing and ideal career [24]. Therefore, the HCC-NCC instrument was developed to follow the concept of the tool which comprised of two set of scales with parallel items. These scales enable the comparison between the influences of healthcare career choice and perception of nursing as a career choice. Fifty parallel items were formulated for each scale, with seven to nine items in each subscale. All items were derived from the participants’ words during the focus group discussions, broad literature review including existing instruments, and the developers’ clinical experience and inferential reasoning. A large pool of items was selected and sampled systematically to include all the content that could be potentially relevant to the target construct. This allowed some items to be deleted, considering that subsequent psychometric analyses could potentially identify weak and unrelated items [30]. All items were developed to be rated on a five-point Likert rating scale (1 = strongly disagree, 2 = disagree, 3 = neutral, 4 = agree, 5 = strongly agree), with higher scores indicating more influential career attribute and lesser scores indicating less influential career attribute.

#### Content validation

Twelve content experts were invited to evaluate the content validity of the initial 50 instrument. These experts included three nursing educators from three restructured hospitals, seven nursing and two allied health lecturers from two higher educational institutions, and two health administrators from the Ministry of Health. The experts independently reviewed each item using the 4-point rating scale (1 = not relevant, 2 = somewhat relevant, 3 = quite relevant, 4 = very relevant). Content validity index (CVI) was computed for each item by the number of experts giving a rating of either 3 or 4 and divided by the overall number of overall experts. Three items with CVI <0.75 were removed as they were identified as being vague or similar to other items [31]. Five more items were added based on the experts’ recommendations. The revised instrument was sent for a second round of content validation which yielded at least a CVI of 0.78 for each item, a CVI of 0.78 for the HCC, and a CVI of 1.0 for the NCC scale.

#### Pilot testing

A pilot test was conducted with 15 medical students and 15 nursing students to establish face validity of the instrument, consisting of 52-parellel items. This pilot testing aimed to: (1) evaluate the clarity of each item and instructions provided, (2) receive feedback about the format of the tool, (3) find out the time taken to complete the instrument, and (4) perform a preliminary check on the internal consistency of the scales. The participants did not express any difficulty with the wording and format of the instrument. They reported taking approximately 15–20 min to complete the instrument. The preliminary Cronbach’s alpha of the instrument from the pilot test was reported to be 0.72 to 0.94.

### Phase 2: Psychometric evaluation of the instrument

A psychometric testing was conducted to evaluate the psychometric properties of the newly developed HCC-NCC instrument, including factor structure, internal consistency, test-retest reliability, and concurrent validity.

#### Setting and participants

A convenience sampling method was used to recruit students from one university and one polytechnic institution in Singapore. All first-year students, between 16 and 25 years old and undertaking healthcare related courses at the two institutions, were invited to participate in the study. The initial 52-item HCC-NCC instrument was administered to a total of 300 participants. The sample size of 300 participants was based on Gorsuch’s recommendation of a minimum of 5 respondents per item, and a probability of receiving incomplete questionnaires [32]. There were 283 completed questionnaires.

#### Data collection and procedure

Following approval from a University Institutional Review Board, email invitations with link to the survey questionnaires were sent out to the potential participants between July to August 2015. The questionnaires included the initial 52-item HCC-NCC instrument and the Indiana Instrument with 17 parallel items. The content validity of the Indiana Instrument was established by a panel of experts and contents from the literature reviews, with the Cronbach’s alpha reported to be 0.84 for the ideal career and 0.81 for the nursing career [18]. About two weeks after the completion of the questionnaires, participants were invited to complete the parallel scales for a second time, to establish the stability of the instrument. The two weeks’ time interval would be lengthy enough for respondents to be unable to remember their original responses, and yet not too long for their attitudes of the material to have changed [33].

#### Data analysis

Descriptive statistics were computed for demographic variables. Construct validity was assessed by an exploratory factor analysis using the principal component analysis and varimax rotation to examine the factor construct of the instrument. The number of factors were determined by eigenvalues >1. Concurrently validity was tested by Pearson’s correlation coefficients. Internal consistency was evaluated using Cronbach’s alpha and item-to-total correlation. Test-retest reliability was assessed using Intraclass Correlation Coefficient (ICC).

## Results

The demographic characteristics of the 283 participants are presented in Table 1. The students were between the age of 16 and 25, with a mean age of 19 (SD = 1.79). The majority of them were female (*n* = 224, 79.2%) and nursing students (*n* = 175, 61.8%).

**Table 1:** Summary of demographic characteristics of healthcare students

Characteristic	N	%
Age		
16–20	210	74.2
21–25	73	25.8
Gender		
Male	59	20.8
Female	224	79.2
Course (Non-nursing)	108	38.2
Bachelor of Medicine	16	5.6
Bachelor of Science (Pharmacy)	56	19.8
Diploma in Physiotherapy	14	5
Diploma in Occupational Therapy	12	4.2
Diploma in Dental Hygiene Therapy	5	1.8
Diploma in Social Work	5	1.8
Course (Nursing)	175	61.8
Bachelor of Science (Nursing)	25	8.8
Diploma of Nursing	150	53
Relatives in healthcare professions		
No	188	66.4
Yes	95	33.6

### Construct validity

Exploratory factor analysis was used to examine the factor structure of both HCC-NCC parallel scales. Bartlett’s test of sphericity was statically significant for the HCC (×2 (595) = 5161.30, *p* < 0.001) and NCC (×2 (595) = 6344.80, *p* < 0.001). The Kaiser-Meyer-Olkin measure of sampling adequacy was 0.90 for the HCC and 0.91 for the NCC scale, indicating that the sample was large enough to perform factor analysis.

**Fig. 1:**
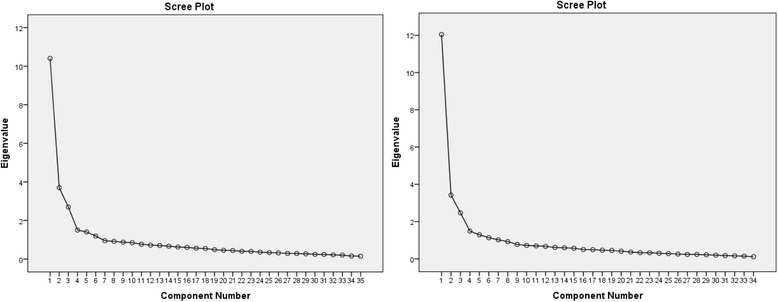
Scree plot for HCC scale (*left*) & NCC scale (*right*)

The initial principal component analysis (PCA) showed that all 52 items extracted a eleven-factor solution for HCC and nine-factor solution for NCC using eigenvalue >1. The factor analysis using varimax rotation was then conducted. Items with factor loading <0.4 or items that loads equally on two factors were removed. Any item removed for HCC was also removed for NCC to ensure consistency in keeping the items in both scales parallel to each other. A total of 17 items were removed with this procedure. Using PCA with eigenvalues >1, six factors were extracted from the remaining 36 items which accounted for 59 and 64% of the variance for the HCC and NCC scales respectively. The scree plots in Fig. 1 illustrated the number of factors.

Tables 2 and 3 summarises the result of the rotated component matrix using varimax rotation. Using the loading criteria of 0.40 [34], 35 items demonstrated strong factor loadings ranging from 0.48 to 0.82 for the HCC and 0.41 to 0.84 for the NCC scale. Factor 1, personal interest, consisted of five items with factor loadings of 0.57 to 0.84 and accounted for 12.60 and 12.89% of the variance for the HCC and NCC scales respectively. Factor 2, prior healthcare exposure, consisted of six items with factor loadings of 0.55 to 0.78 and accounted for 12.11% (HCC) and 12.52% (NCC) of the variance. Factor 3, self-efficacy, composed of four items with factor loadings of 0.49 to 0.73 and accounted for 10.71% (HCC) and 11.97% (NCC) of the variance. Factor 4, perceived nature of work, consisted of six items with factor loadings of 0.41 to 0.77 and accounted for 10.21% (HCC) and 10.99% (NCC) of the variance. Factor 5, job prospects, consisted of seven items with factor loadings of 0.44 to 0.79 and accounted for 7.78% (HCC) & 8.12% (NCC) of the variance. Factor 6, social influences, consisted of eight items with factor loadings of 0.45 to 0.79 and accounted for 6.32% (HCC) & 7.58% (NCC) of variance. This six factor solutions, therefore, represented the core subscales of the instrument.

**Table 2:** Principal component analysis with Varimax rotation of the HCC scale

		Factor loadings					
Item	Healthcare career choice	1	2	3	4	5	6
1	I desire to help others	0.059	0.047	0.795	0.138	0.107	−0.053
2	I can contribute to the society	0.033	0.103	0.817	0.082	0.132	−0.025
3	I desire for a fulfilling career	−0.01	0.008	0.775	−0.02	0.095	0.085
6	I enjoy interacting with people	0.174	−0.07	0.565	0.339	0.114	0.045
7	I want to make a difference in someone’s life	0.096	0.018	0.813	0.071	0.119	−0.012
9	In taking care of a sick family member	0.166	0.169	0.023	0.609	0.03	0.222
10	In being taken care of by a healthcare professional	0.025	0.075	−0.01	0.736	0.16	0.163
11	In my school co-curriculum activities	0.053	0.226	−0	0.67	0.113	0.069
12	In observing a healthcare professional at work	−0.108	0.218	0.262	0.597	0.127	0.095
13	In hearing about the profession from significant others	0.181	0.374	0.132	0.551	0.015	0.05
14	Doing voluntary work in healthcare settings	0.076	0.225	0.214	0.71	0.098	0.052
17	It reflects well of my academic ability	0.307	0.192	−0.02	0.272	−0.027	0.596
21	I want to choose a course that is more deserving of my good grades	0.153	0.259	−0.04	0.202	−0.073	0.699
23	I can make autonomous decisions at work	0.085	0.094	0.045	0.142	0.308	0.733
24	I want to be able to make diagnosis	0.066	0.166	0.127	0.104	0.447	0.486
28	It is a highly skilled occupation	0.354	−0.03	0.342	0.074	0.511	0.279
29	I want a more hands-on job	0.097	0.028	0.462	−0.118	0.519	0.07
30	It is a challenging job	0.112	0.128	0.377	0.133	0.655	0.114
32	It is a demanding job	0.212	0.179	0.119	0.256	0.645	0.076
33	I do not mind attending to others’ hygiene needs	0.141	0.323	0.114	0.19	0.549	−0.071
35	It ensures a stable job	0.736	0.176	0.253	−0.109	0.139	0.092
36	I will never be unemployed	0.655	0.1	0.147	−0.08	−0.067	0.295
37	It ensures high income	0.597	0.411	−0.04	0.079	−0.047	0.236
38	The career ensures me a good standard of living	0.743	0.318	0.117	0.068	0.07	0.108
39	It provides a chance to work overseas	0.671	0.196	−0.16	0.222	0.359	0.015
40	It provides many opportunities for my career advancement	0.785	0.261	0.066	0.181	0.238	−0.074
41	It provides a chance to achieve higher qualifications	0.71	0.16	0.04	0.217	0.308	0.114
43	I will be well respected	0.358	0.681	0.059	0.181	0.048	0.188
44	It has good public image	0.313	0.728	−0.06	0.135	0.157	0.205
45	The social media has inspired me	0.151	0.66	−0.07	0.317	0.259	0.108
46	There is no gender stigma in this career	0.28	0.58	−0.03	0.271	0.244	0.01
47	My parents are supportive	0.23	0.477	0.235	0.061	0.257	-0.009
49	I want my parents to be proud of me	0.316	0.555	0.073	0.112	0.247	0.035
51	My peers encouraged me of my choice	0.029	0.584	0.11	0.364	−0.148	0.15
52	My peers will look up to me	0.119	0.718	−0.01	0.285	0.029	0.294

**Table 3:** Principal component analysis with Varimax rotation of the NCC scale

		Factor loadings					
Item	Perception of nursing career	1	2	3	4	5	6
1	Nurses desire to help others	0.177	0.073	0.184	0.838	0.12	0.032
2	Nurses can contribute to the society	0.264	0.039	0.182	0.741	0.183	−0.117
3	Nurses desire for a fulfilling career	0.335	0.172	0.265	0.585	0.189	−0.073
6	Nurses enjoy interacting with people	0.034	0.195	0.207	0.76	0.17	0.084
7	Nurses want to make a difference in someone’s life	0.176	0.156	0.151	0.765	0.275	−0.061
9	In taking care of a sick family member	0.175	0.13	0.613	0.209	−0.016	0.167
10	In being taken care of by a nurse	0.067	0.109	0.78	0.113	−0.001	0.182
11	In their school co-curriculum activities	0.07	0.05	0.727	0.162	0.002	0.16
12	In observing a nurse at work	0.12	0.309	0.717	0.162	0.129	0.151
13	In hearing about the nursing profession from significant others	0.156	0.246	0.683	0.141	0.247	0.082
14	Doing voluntary work in healthcare settings	0.028	0.186	0.722	0.149	0.237	0.065
17	Nursing career reflects well of one’s academic ability	0.071	0.354	0.287	−0.144	−0.068	0.658
21	Nurses want to choose a course that is more deserving of their good	0.083	0.118	0.222	−0.194	0.05	0.684
23	Nurses can make autonomous decisions at work	0.205	0.087	0.155	0.153	0.107	0.729
24	Nurses are able to make diagnosis	0.296	0.207	0.229	0.047	0.152	0.561
28	Nursing is a highly skilled occupation	0.42	0.173	0.17	0.246	0.63	−0.03
29	Nursing is a hands-on job	0.238	0.11	0.168	0.355	0.65	−0.126
30	Nursing is a challenging job	0.225	0.057	0.127	0.308	0.767	0.067
32	Nursing is a demanding job	0.159	0.186	0.044	0.089	0.747	0.201
33	Nurses do not mind attending to others’ hygiene needs	0.02	0.287	0.07	0.375	0.41	0.249
35	Nursing ensures a stable job	0.696	0.052	0.148	0.263	0.323	0.065
36	Nurses will never be unemployed	0.66	0.094	0.071	0.287	0.092	0.135
37	Nurses have high income	0.437	0.481	−0.14	0.048	−0.016	0.439
38	Nursing career ensure a good standard of living	0.639	0.402	0.074	−0.05	0.14	0.265
39	Nurses can work overseas	0.756	0.25	0.128	0.089	0.151	0.101
40	Nurses have many opportunities for my career advancement	0.773	0.218	0.167	0.197	0.135	0.175
41	Nursing career provide a chance to achieve higher qualifications	0.74	0.237	0.142	0.153	0.23	0.183
43	Nurses are well respected	0.26	0.698	0.152	0.242	0.178	0.068
44	Nurses have good public image	0.208	0.724	0.121	0.188	0.143	0.098
45	The social media has inspired them to take up nursing	0.127	0.606	0.174	0.126	0.132	0.25
46	There is no gender stigma in nursing	0.335	0.541	0.039	0.13	−0.03	0.38
47	Their parents are supportive	0.297	0.486	0.398	0.126	0.146	−0.02
49	Nurses want their parents to be proud of them	0.381	0.454	0.363	0.226	0.057	0.022
51	Their peers encouraged them to be nurses	0.147	0.638	0.376	−0.024	0.085	0.184
52	Their peers look up to them as nurses	0.074	0.785	0.254	0.055	0.123	0.121

### Concurrent validity

There was a moderately strong significant positive correlation in the total scores between the Indiana and the HCC-NCC instruments (*r* = 0.76, *p* < 0.001). There were significant positive correlations between the Indiana’s nursing career scales and NCC scale (*r* = 0.73, *p* < 0.001) and between the Indiana’s ideal career scale and HCC scale (*r* = 0.61, *p* < 0.001).

### Internal consistency and inter-item correlations

Table 4 present the results for internal consistency and inter-item correlations. The Cronbach’s alpha of the six subscales ranged from 0.71 to 0.89 for the HCC scale and 0.78 to 0.89 for the NCC scale. The correlation coefficients between items and their respective subscales ranged between 0.39 to 0.78 for the HCC scale and 0.46 to 0.80 for the NCC scale. The overall Cronbach’s alpha for all 35 items was 0.93 for the HCC and 0.94 for the NCC scale.

**Table 4:** Scale item statistics

Item	Cronbach’s alpha	Item-total correlation
Healthcare career choice		
Factor 1: Personal Interest	0.847	
1﻿ I desire to help others		0.711
2 I can contribute to the society		0.732
3 I desire for a fulfilling career		0.627
6 I enjoy interacting with people		0.515
7 I want to make a difference in someone’s life		0.716
Factor 2: Prior Healthcare Exposure	0.812	
9 In taking care of a sick family member		0.534
10 In being taken care of by a healthcare professional		0.59
11 In my school co-curriculum activities		0.552
12 In observing a healthcare professional at work		0.555
13 In hearing about the profession from significant others		0.559
14 Doing voluntary work in healthcare settings		0.659
Factor 3: Self efficacy	0.712	
17 It reflects well of my academic ability		0.51
21 Iwant to choose a course that is more deserving of my good grades		0.538
23 I can make autonomous decisions at work		0.577
24 I want to be able to make diagnosis		0.388
Factor 4: Perceived nature of work	0.775	
28 It is a highly skilled occupation		0.557
29 I want a more hands-on job		0.485
30 It is a challenging job		0.679
32 It is a demanding job		0.553
33 I do not mind attending to others’ hygiene needs		0.484
Factor 5: Job prospects	0.886	
35 It ensures a stable job		0.682
36 I will never be unemployed		0.56
37 it ensures high income		0.614
38 The career ensures me a good standard of living		0.752
39 It provides a chance to work overseas		0.656
40 it provides many opportunities for my career advancement		0.781
41 It provides a chance to achieve higher qualifications		0.707
Factor 6: Social influences	0.873	
43 I will be well respected		0.714
44 It has a good public image		0.744
45 The social media has inspired me		0.701
46 There is no gender stigma in this career		0.626
47 My parents are supportive		0.472
49 I want my parents to be proud of me		0.58
51 My peers encouraged me of my choice		0.501
52 My peers will look up to me		0.722
Perception of nursing career		
Factor 1: Personal interest	0.889	
1 Nurses desire to help others		0.781
2 Nurses can contribute to the society		0.751
3 Nurses desire for a fulfilling career		0.667
6 Nurses enjoy interacting with people		0.686
7 Nurses want to make a difference in someone’s life		0.774
Factor 2: Prior healthcare exposure	0.869	
9 In taking care of a sick family member		0.576
10 In being taken care of by a nurse		0.713
11 In their school co-curriculum activities		0.634
12 In observing a nurse at work		0.739
13 in hearing about the nursing profession from significant others		0.694
14 Doing voluntary work in healthcare settings		0.669
Factor 3: Self efficacy	0.776	
17 Nursing career reflects well of one’s academic ability		0.625
21 Nurses want to choose a course that is more deserving of their good grades		0.577
23 Nurses can make autonomous decisions at work		0.58
24 Nurses are able to make diagnosis		0.633
Factor 4: Perceived nature of work	0.823	
28 Nursing is a highly skilled occupation		0.669
29 Nursing is a more hands-on job		0.634
30 Nursing is a challenging job		0.747
32 Nursing is a demanding job		0.602
33 Nurses do not mind attending to others’ hygiene needs		0.464
Factor 5: Job prospects	0.893	
35 Nursing ensures a stable job		0.679
36 Nurses will never be unemployed		0.639
37 Nurses have high income		0.549
38 Nursing career ensures a good standard of living		0.712
39 Nurses can work overseas		0.72
40 Nurses have many opportunities for my career advancement		0.801
41 Nursing career provide a chance to achieve higher qualifications		0.777
Factor 6: Social influences	0.881	
43 Nurses are well respected		0.714
44 Nurses have a good public image		0.699
45 The social media has inspired them to take up nursing		0.6
46 There is no gender stigma in thhis nursing		0.584
47 Their parents are supportive		0.6
49 Nurses want their parents to be proud of them		0.607
51 Their peers encouraged them to be nurses		0.645
52 Their peers will look up to them as nurses		0.731

### Test-retest reliability

Twenty-eight participants completed the HCC-NCC instrument for the second time after two weeks’ interval. The ICC was 0.63 (95% CI = 0.267–0.813, *p* = 0.002) for HCC and 0.60 (95% CI = 0.206–0.798, *p* = 0.005) for NCC.

## Discussion

The competition among healthcare courses to attract high quality school leavers is becoming increasingly intense. This is particularly challenging in the nursing course which requires the largest recruitment target to meet the workforce demand [35]. Using the Indiana Instrument, most studies to date have primarily focused on school students about the differences in attitudes between ‘ideal career’ and ‘nursing as a career’ [25, 27]. By applying the parallel scales concept of the Indiana Instrument, we developed and tested the HCC-NCC instrument in order to compare the influences of healthcare career choice and perception of nursing as a career choice among the healthcare students.

The content validity of the HCC-NCC instrument was achieved through a combination of literature review, findings from a previous qualitative study, and experts’ validation. Inclusion of a total of 12 experts from a variety of settings including education institutions, hospitals and the Ministry of Health provided a wide perspective of the tool. Based on the experts’ validation, the CVI achieved Lynn’s (1986) criterion for content validity [36]. Additionally, a pilot test with 30 non-experts, established the face validity.

The construct validity of the HCC-NCC instrument was assessed in a factor analysis by using a principal component analysis. Factor analysis was justified with Bartlett’s test of sphericity, while the calculated Kaiser-Meyer-Olkin measure of sampling adequacy indicated that there was adequate sample for factor analysis [37]. Modifications to the instruments were made following the factor analysis to remove items that were found to be weak or unrelated. Finally, all included items possessed factor loadings of >0.4 and accounted for 59 and 64% of the variance for the HCC and NCC scales respectively. The factor analysis extracted six factors corresponding with five out of the six career factors that emerged from the findings of a qualitative study [29].

The “personal interest” subscale, refers to the students’ personal interest in their chosen professions. According to Holland’s theory of “Career Typology,” individuals choose career environments that best fit their personality and interest [38]. Several studies have shown that students pursuing healthcare careers tend to have similar interests [5, 7, 39]. A personal interest in their chosen professions based on notions of altruism, opportunity to interact with others, as well as an interest for science-related subjects were expressed among the healthcare students in a previous study [29].

The second factor, “prior healthcare exposure”, reflects how healthcare related experiences could influence their choice of a healthcare career, both positively and negatively. The influence in the developmental stage on career choice, spanning from school years to young adulthood, has long been established by a vocational psychologist [40, 41]. The exposure of students to healthcare-related work, including observing a healthcare professional at work has shown to draw students into a healthcare career [42, 43].

The third factor, “self-efficacy”, refers to a set of self-beliefs about one’s personal competence to perform the actions required to produce outcomes in particular domains [44]. Applying the social cognitive career theory, the links between self-efficacy and career choice has been well-established by Lent et al. [45]. Academic ability which often reflects intelligence serves as an important indicator for an individual to evaluate one’s self-efficacy to an academic related career choice. Nursing is often perceived as a course for students with low academic ability which could have deterred academically-abled students from joining the course [46].

The factor, “job prospects”, considers the practical aspects of a healthcare career that could influence the career choice. This includes a desire for job opportunity, job stability, and good income. Healthcare careers are often highly regarded for the ease of getting a job and job stability [10, 11]. Nursing is however often perceived as a poorly paid job [23].

The factor, “perceived nature of work”, relates to how students’ perceived the characteristics of the healthcare careers that influenced their choice of career. The characteristics associated with nursing work, including the involvement of too much hard work [21], and ‘dirty’ work [22] have deterred students from joining the nursing profession.

The final factor, “social influences”, includes social status, gender-type and significant others that have been found to have a significant impact on the students’ career decision-making process. Social influences by significant others was found to affect the career aspirations of Asian students more significantly than the Western students [47, 48].

The concurrent validity of the HCC-NCC instrument was examined by correlating with the Indiana Instrument, both of which were administered at the same time. The Indiana Instrument is one of the most widely used tool for determining the difference between the ideal career and a nursing career with tested reliability and validity [24]. A significant strong positive correlation was found between these two scales, confirming the concurrent validity of the HCC_NCC instrument.

Besides evidence to support the validity of the HCC-NCC scales, the study demonstrated a satisfactory internal consistency as reflected by the Cronbach’s alpha of 0.71 to 0.89 for both scales and its subscale, and the high correlation between the items with their respective subscales. The stability of the HCC-NCC instrument was also demonstrated.

In comparison to the existing 17-parallel items Indiana Instrument, the 35-parallel items HCC-NCC instrument offer a more comprehensive comparison of career choice influences. The instrument has many potential applications for future use. The instrument provides a comparison of factors influencing healthcare career choice and perception of nursing as a career. Such comparison can highlight the differences between career influences in non-nursing careers and a nursing career which has the potential to identify specific strategies to enhance nursing recruitment. Each of the parallel scales can also be used as a stand-alone scale. While the HCC scale can be used for identifying factors influencing healthcare career choice, the NCC scale can be used for examining factors influencing nursing as a career choice. Findings obtained from the instrument may aid in the development of recruitment programmes. The effectiveness of the recruitment interventions can be evaluated using this instrument in a pretest-posttest study or in randomised-controlled trials. Future studies may consider adapting the HCC scale for graduating students to examine influences of career choice on healthcare sub-specialities.

### Limitations

Convenience sampling was used to recruit participants in this study, which may lead to potential biasness such as under-representation of different targeted groups within the sample. Future studies with a larger sample size and random sampling may lend added support to the validity and reliability of the instrument. While an acceptable test- retest reliability is achieved, it could be strengthened through a larger sample size of participants attempting the retest after two weeks.

## Conclusions

This study has developed and established valid and reliable 35-item instrument with six career-choice factors to compare healthcare career choice and nursing as a career choice (See Additional file 1). The HCC-NCC instrument proves useful for future studies to determine how strongly each of the factors is associated with the healthcare career choice and with nursing as a career, and to examine the differences between students’ healthcare career choice and their perception of nursing as a career choice. The findings may have potential implications for educational institutions and policy-making planners to consider specific recruitment nursing strategies.

## Additional file

Additional file 1: HCC_NCC Questionnaire. (DOCX 30 kb)

## Abbreviations

CVI: Content validity index
HCC: Healthcare career choice
ICC: Intraclass Correlation Coefficient
NCC: Nursing Career Choice
