# Supplementary material for: Development and psychometric testing of an instrument to compare career choice influences and perceptions of nursing among healthcare students
Source: BMC Med Educ. 2017 Apr 27;17:72. doi: 10.1186/s12909-017-0910-7 (PMC5408422; doi:10.1186/s12909-017-0910-7)
Supplement: Additional file 1: — HCC_NCC Questionnaire. (DOCX 30 kb) [file 12909_2017_910_MOESM1_ESM.docx]

**Healthcare Career Choice (HCC) Questionnaire**

How do you feel about the following statements with regards to your healthcare career choice?

Please *circle* the number that best describe how you feel.

|  | *Strongly Disagree* | *Disagree* | *Neutral* | *Agree* | *Strongly Agree* |
| --- | --- | --- | --- | --- | --- |
|  |  |  |  |  |  |
| **I have made my current career choice because________** |  |  |  |  |  |
| 1. I desire to help others | *1* | *2* | *3* | *4* | *5* |
| 1. I can contribute to the society | *1* | *2* | *3* | *4* | *5* |
| 1. I desire for a fulfilling career | *1* | *2* | *3* | *4* | *5* |
| 1. I enjoy interacting with people | *1* | *2* | *3* | *4* | *5* |
| 1. I want to make a difference in someone’s life | *1* | *2* | *3* | *4* | *5* |
|  |  |  |  |  |  |
|  |  |  |  |  |  |
| **I have made my current career choice because of my experience________** |  |  |  |  |  |
| 1. In taking care of a sick family member | *1* | *2* | *3* | *4* | *5* |
| 1. In being taken care of by a healthcare professional | *1* | *2* | *3* | *4* | *5* |
| 1. In my school co-curriculum activities (E.g Red cross, St John’s etc.) | *1* | *2* | *3* | *4* | *5* |
| 1. In observing a healthcare professional at work | *1* | *2* | *3* | *4* | *5* |
| 1. In hearing about the profession from significant others | *1* | *2* | *3* | *4* | *5* |
| 1. Doing voluntary work in healthcare settings | *1* | *2* | *3* | *4* | *5* |
|  |  |  |  |  |  |
|  | *Strongly Disagree* | *Disagree* | *Neutral* | *Agree* | *Strongly Agree* |
|  |  |  |  |  |  |
| **I have made my current career choice because________** |  |  |  |  |  |
|  |  |  |  |  |  |
| 1. It reflects well of my academic ability | *1* | *2* | *3* | *4* | *5* |
| 1. I want to choose a course that is more deserving of my good grades | *1* | *2* | *3* | *4* | *5* |
| 1. I can make autonomous decisions at work | *1* | *2* | *3* | *4* | *5* |
| 1. I want to be able to make diagnosis | *1* | *2* | *3* | *4* | *5* |
|  |  |  |  |  |  |
|  |  |  |  |  |  |
| **I have made my current career choice because________** |  |  |  |  |  |
|  |  |  |  |  |  |
| 1. It is a highly skilled occupation | *1* | *2* | *3* | *4* | *5* |
| 1. I want a more hands-on job | *1* | *2* | *3* | *4* | *5* |
| 1. It is a challenging job | *1* | *2* | *3* | *4* | *5* |
| 1. It is a demanding job | *1* | *2* | *3* | *4* | *5* |
| 1. I do not mind attending to others’ hygiene needs | *1* | *2* | *3* | *4* | *5* |
|  |  |  |  |  |  |
|  |  |  |  |  |  |
|  |  |  |  |  |  |
| **I have made my current career choice because________** |  |  |  |  |  |
|  |  |  |  |  |  |
| 1. It ensures a stable job | *1* | *2* | *3* | *4* | *5* |
| 1. I will never be unemployed | *1* | *2* | *3* | *4* | *5* |
| 1. It ensures high income | *1* | *2* | *3* | *4* | *5* |
| 1. The career ensures me a good standard of living | *1* | *2* | *3* | *4* | *5* |
| 1. It provides a chance to work overseas | *1* | *2* | *3* | *4* | *5* |
| 1. It provides many opportunities for my career advancement | *1* | *2* | *3* | *4* | *5* |
| 1. It provides a chance to achieve higher qualifications | *1* | *2* | *3* | *4* | *5* |
|  |  |  |  |  |  |
|  |  |  |  |  |  |
|  |  |  |  |  |  |
|  |  |  |  |  |  |
|  | *Strongly Disagree* | *Disagree* | *Neutral* | *Agree* | *Strongly Agree* |
| **I have made my current career choice because________** |  |  |  |  |  |
|  |  |  |  |  |  |
| 1. I will be well respected | *1* | *2* | *3* | *4* | *5* |
| 1. It has a good public image | *1* | *2* | *3* | *4* | *5* |
| 1. The social media has inspired me | *1* | *2* | *3* | *4* | *5* |
| 1. There is no gender stigma in this career | *1* | *2* | *3* | *4* | *5* |
| 1. My parents are supportive |  |  |  |  |  |
| 1. I want my parents to be proud of me | *1* | *2* | *3* | *4* | *5* |
| 1. My peers encouraged me of my choice | *1* | *2* | *3* | *4* | *5* |
| 1. My peers will look up to me | *1* | *2* | *3* | *4* | *5* |

**Nursing as a Career Choice (NCC) Questionnaire**

How do you feel about the following statements with regards to perception of nursing as a career choice?

Please *circle* the number that best describe how you feel.

|  | *Strongly Disagree* | *Disagree* | *Neutral* | *Agree* | *Strongly Agree* |
| --- | --- | --- | --- | --- | --- |
| **Nurses made their career choice**  **because** __________ |  |  |  |  |  |
| 1. Nurses desire to help others | *1* | *2* | *3* | *4* | *5* |
| 1. Nurses can contribute to society | *1* | *2* | *3* | *4* | *5* |
| 1. Nurses desire for a fulfilling career | *1* | *2* | *3* | *4* | *5* |
| 1. Nurses enjoy interacting with people | *1* | *2* | *3* | *4* | *5* |
| 1. Nurses want to make a difference in someone’s life | *1* | *2* | *3* | *4* | *5* |
|  |  |  |  |  |  |
|  |  |  |  |  |  |
| **Nurses made their career choice**  **because of their experience__________** |  |  |  |  |  |
| 1. In taking care of a sick family member | *1* | *2* | *3* | *4* | *5* |
| 1. In being taken care of by a nurse | *1* | *2* | *3* | *4* | *5* |
| 1. In their school co-curriculum activities (E.g Red Cross, St John’s etc.) | *1* | *2* | *3* | *4* | *5* |
| 1. In observing a nurse at work | *1* | *2* | *3* | *4* | *5* |
| 1. In hearing about the nursing profession from significant others | *1* | *2* | *3* | *4* | *5* |
| 1. Doing voluntary work in healthcare settings | *1* | *2* | *3* | *4* | *5* |
|  |  |  |  |  |  |
|  |  |  |  |  |  |
| **Nurses made their career choice**  **because __________** |  |  |  |  |  |
| 1. Nursing career reflects well of one’s academic ability | *1* | *2* | *3* | *4* | *5* |
| 1. Nurses want to choose a course that is more deserving of their good grades | *1* | *2* | *3* | *4* | *5* |
| 1. Nurses can make autonomous decisions at work | *1* | *2* | *3* | *4* | *5* |
| 1. Nurses are able to make diagnosis | *1* | *2* | *3* | *4* | *5* |
|  |  |  |  |  |  |
|  |  |  |  |  |  |
| **Nurses made their career choice**  **because __________** | *Strongly Disagree* | *Disagree* | *Neutral* | *Agree* | *Strongly Agree* |
| 1. Nursing is a highly skilled occupation | *1* | *2* | *3* | *4* | *5* |
| 1. Nursing is a hands-on job | *1* | *2* | *3* | *4* | *5* |
| 1. Nursing is a challenging job | *1* | *2* | *3* | *4* | *5* |
| 1. Nursing is a demanding job | *1* | *2* | *3* | *4* | *5* |
| 1. Nurses do not mind attending to other’s hygiene needs | *1* | *2* | *3* | *4* | *5* |
|  |  |  |  |  |  |
|  |  |  |  |  |  |
| **Nurses made their career choice**  **because __________** |  |  |  |  |  |
|  |  |  |  |  |  |
| 1. Nursing ensures a stable job | *1* | *2* | *3* | *4* | *5* |
| 1. Nurses will never be unemployed | *1* | *2* | *3* | *4* | *5* |
| 1. Nurses have high income | *1* | *2* | *3* | *4* | *5* |
| 1. Nursing career ensure a good standard of living | *1* | *2* | *3* | *4* | *5* |
| 1. Nurses can work overseas | *1* | *2* | *3* | *4* | *5* |
| 1. Nurses have many opportunities for career advancement | *1* | *2* | *3* | *4* | *5* |
| 1. Nursing career provide a chance to achieve higher qualifications | *1* | *2* | *3* | *4* | *5* |
|  |  |  |  |  |  |
|  |  |  |  |  |  |
| **Nurses made their career choice**  **because *___________*** |  |  |  |  |  |
| 1. Nurses are well respected | *1* | *2* | *3* | *4* | *5* |
| 1. Nurses have good public image | *1* | *2* | *3* | *4* | *5* |
| 1. The social media has inspired them to take up nursing | *1* | *2* | *3* | *4* | *5* |
| 1. There is no gender stigma in nursing | *1* | *2* | *3* | *4* | *5* |
| 1. Their parents are supportive |  |  |  |  |  |
| 1. Nurses want their parents to be proud of them | *1* | *2* | *3* | *4* | *5* |
| 1. Their peers encouraged them to be nurses | *1* | *2* | *3* | *4* | *5* |
| 1. Their peers look up to them as nurses | *1* | *2* | *3* | *4* | *5* |
